# CPG-Based Control of an Octopod Biomimetic Machine Lobster for Mining Applications: Design and Implementation in Challenging Underground Environments

**DOI:** 10.3390/s25144331

**Published:** 2025-07-11

**Authors:** Jianwei Zhao, Haokun Zhang, Mingsong Bao, Boxiang Yin, Yiteng Zhang, Zhen Jiang

**Affiliations:** 1 Faculty of Mechanical and Electrical Engineering, China University of Mining and Technology (Beijing), Beijing 100083, China; 18731415524@163.com (H.Z.); a13765252515@163.com (Z.J.); 2 Shandong Guoxing Intelligent Technology Co., Ltd., Yantai 264000, China; terrybao@suprobot.com; 3 School of Electrical and Electronic Engineering, College of Engineering, Nanyang Technological University, 50 Nanyang Avenue, Singapore 639798, Singapore; boxiang002@e.ntu.edu.sg; 4 Emergency Management Department Information Research Institute, Beijing 100029, China; zhangyt1996@163.com

**Keywords:** octopod bionic robot, shape memory alloys, CPG control, Kuramoto model, gait design

## Abstract

Central pattern generators (CPGs) have been extensively researched and validated as a well-established methodology for bionic control, particularly within the field of legged robotics. However, investigations concerning octopod robots remain relatively sparse. This study presents the design of an octopod robotic system inspired by the biological characteristics of lobsters. The machine lobster utilizes remote sensing technology to execute designated tasks in subterranean and mining environments, with its motion regulated by CPGs, accompanied by a comprehensive simulation analysis. The research commenced with the modeling of a biomimetic lobster robot, which features a three-degree-of-freedom leg structure and torso, interconnected by shape memory alloys (SMAs) that serve as muscle actuators. Mathematically, both forward and inverse kinematics were formulated for the robot’s legs, and a 24-degree-of-freedom (DOF) gait pattern was designed and validated through MATLAB 2020a simulations. Subsequently, a multi-layer mesh CPG neural network model was developed utilizing the Kuramoto model, which incorporated frustration effects as the rhythm generator. The control model was constructed and evaluated in Simulink, while dynamic simulations were conducted using Adams 2022 software. The findings demonstrate the feasibility, robustness, and efficiency of the proposed CPG network in facilitating the forward locomotion of the lobster robot, thereby broadening the range of control methodologies applicable to octopod biomimetic robots.

## 1. Introduction

Traditional wheeled, tracked, and industrial robots are primarily limited to executing simple and repetitive tasks within structured industries, which are characterized by straightforward working conditions and a minimal risk of accidents [1]. These conventional robotic systems are inadequately equipped to operate effectively in unstructured environments, which are defined by complex terrains and ambiguous objectives. In response to the limitations of traditional robotic systems, bionic robots have been developed [2]. These robots, which emulate the movements of living organisms, tend to exhibit superior performance in complex geographic settings that involve intricate and poorly defined tasks, such as search operations following natural disasters, including floods, fires, and earthquakes, as well as underwater search and retrieval missions. Among the various types of biomimetic robots, footed robots are the most widely utilized. Due to the high degrees of freedom in their legs, footed robots demonstrate enhanced overall steering and locomotion flexibility, enabling them to navigate obstacles and perform tasks in complex geographic environments [3]. Within the category of footed robots, quadrupedal and hexapod robots have a broader range of applications. Since the development of a hydraulically driven quadrupedal robot by Carnegie Mellon University in 1986 [4], quadrupedal robots have undergone significant advancements. In 2006, Boston Dynamics introduced a quadrupedal robotic dog named BigDog, which made notable progress in weight-bearing capacity, walking ability, and endurance [5]. Subsequently, the company released AlphaDog [6], Cheetah [7], and other quadrupedal robotic dogs, which achieved even greater advancements in stability, balance, and robustness. During the 1980s, MIT developed a more sophisticated six-legged robot known as Genghis, which could efficiently navigate complex geographic environments using feedback mechanisms [8]. In 2013, NASA’s Jet Propulsion Laboratory developed ATHLETE, a six-legged robot designed to perform tasks during extraterrestrial landings [9]. The current trend in the development of intelligent and multifunctional legged robots has led to an increased application of these technologies in complex scenarios. Within the field of bionic robot control, the central pattern generator (CPG) control method, which is based on biomimetic principles, has demonstrated significant effectiveness in terms of stability and robustness [10]. The concept of CPG control originates from the study of biological rhythmic phenomena, where the fundamental motor output signals in vertebrates are generated by central pattern generators located in the spinal cord [11]. In recent years, researchers have conducted many studies based on the CPG theory. For example, Fukuoka and Kimura in Japan developed a CPG-based biological control model that enabled a quadrupedal robot to traverse irregular terrains at moderate speeds [12]. Additionally, Ijspeert et al. conducted extensive research on central pattern generators in quadrupedal robots [13], underwater imitation robots modeled after seven-gill eels [14], and amphibious robots resembling salamanders [15], implementing a variety of practical applications. Despite the considerable advancements in research on legged robots, the focus has predominantly been on conventional quadrupedal and hexapod designs, with relatively limited exploration of octopod bionic robots. Grzelczyk et al. proposed the SINE generator, simplifying the control architecture using a sine function and significantly reducing center of gravity fluctuations and energy consumption; however, this generator has not been validated for adaptability in unstructured terrains [16,17]. Yi Zhang et al. [18] designed a disassemblable modular octopedal platform (two quadruped robots connected together) to reduce mechanical complexity. However, their connection mechanism is a rigid joint, limiting body degrees of freedom. Hongzhe Sun et al. developed a closed-chain leg mechanism octopedal robot to validate the feasibility of wheel-foot hybrid locomotion, but the system was overly complex [19]. Jianxu Wu et al. achieved a balance between the simplicity of closed-chain mechanisms and terrain adaptability through trunk-level reconfiguration, offering a new approach for heavy-duty legged platforms; however, their platform’s steering capability was reliant on ground friction [20].

Currently, there are several common CPG models, among which the Matsuoka model [21], Kuramoto model [22,23], and Hopf model [24] are the most representative. The Matsuoka model is based on the nonlinear dynamics of neurons and can generate stable rhythmic signals, but it has complex parameter adjustments and certain limitations in multi-modal motion control. The Hopf model, grounded in Hopf bifurcation theory, can effectively simulate the periodicity and stability of biological movements. However, it is sensitive to initial conditions and faces challenges in achieving synchronization in large-scale networks. The Kuramoto model is a classical phase oscillator model that describes interactions between oscillators through simple phase coupling equations, playing a significant role in the study of synchronization phenomena. Nevertheless, the basic Kuramoto model has a notable deficiency in phase difference control, making it difficult to achieve precise phase control. This is a critical issue for high-degree-of-freedom (DOF) systems and complex coordinated robotic movements. For example, in multi-legged robots, the precise coordination of leg movements is essential for stable locomotion, a requirement that the basic Kuramoto model struggles to meet.

To address the phase difference control limitations of the basic Kuramoto model, researchers have proposed the Kuramoto model with frustration effects. This improved approach introduces frustration effects by adding a non-zero phase shift in the coupling between oscillators, enabling better regulation of phase differences and more precise phase control. This modification partially compensates for the shortcomings of the basic Kuramoto model, opening new possibilities for CPG applications in complex robotic systems [25].

In terms of CPG network structures, there are primarily two types: chain structures and grid structures. Chain structures are simple and intuitive, easy to implement, and suitable for simple rhythmic motion control. However, they have relatively weak parallel control capabilities and slower synchronization convergence in large-scale networks. In contrast, grid structures offer significant advantages. CPG networks with grid structures can achieve better parallel control, allowing multiple oscillators to interact and synchronize simultaneously, which is crucial for complex multi-DOF robotic motion control. Additionally, grid structures excel in fast convergence, quickly reaching stable synchronization states. This is a major advantage for robotic tasks requiring rapid response and coordination. For instance, in the coordinated control of an octopod robot, a grid-structured CPG network can efficiently synchronize the movements of all eight legs, enhancing the robot’s performance and stability.

Therefore, this article presents a comprehensive model of a bionic machine lobster. It employed the Kuramoto model, incorporating frustration effects, as a rhythm generator. It constructed a mathematical model of a multilayer mesh central pattern generator (CPG) neural network, culminating in the development of a CPG neural network model for the machine lobster. The physical design features a three-degree-of-freedom leg structure and torso, which are integrated into a cohesive unit. Memory metal is utilized as a muscle actuator to connect the torso and leg structures. From a mathematical standpoint, the article establishes both the forward and inverse kinematics for the three-degree-of-freedom legs of the machine lobster. Additionally, it devised a 24-degree-of-freedom coupled walking gait along with its mathematical representation. This article aims to utilize remote sensing technology to enable robotic lobsters to execute tasks in subterranean or mining environments.

The feasibility of the mathematical modeling and gait design was validated through simulations conducted in MATLAB, which yielded a feasible region and ultimately determined the size parameters of the machine lobster. Finally, a control model was established using Simulink to simulate the gait of the machine lobster, and gait simulations were also performed using Adams software.

## 2. Machine Lobster Modeling

### 2.1. Physical Modeling of Machine Lobster

Decapod crustaceans such as lobsters face unique challenges due to the complex and variable underwater terrain. Thanks to their multi-degree-of-freedom leg structures, lobsters can navigate through obstacles with high maneuverability, adjusting their direction step by step, performing in-place rotations, and achieving yaw maneuvers through differences in step speed or step amplitude. Their large chelipeds and abdominal structures result in a unique mass distribution, and buoyancy in seawater offsets approximately seven-eighths of their body weight, often making lateral hydrodynamic forces greater than vertical gravitational forces. To address stability issues caused by low effective gravity, lobsters use their chelipeds and abdomen as hydrodynamic control surfaces for pitch stability and deploy their walking legs to provide roll stability. Compared to insects, lobsters have less traction, but they compensate through the evolution of additional legs and active posture adjustments.

**Figure 1:**
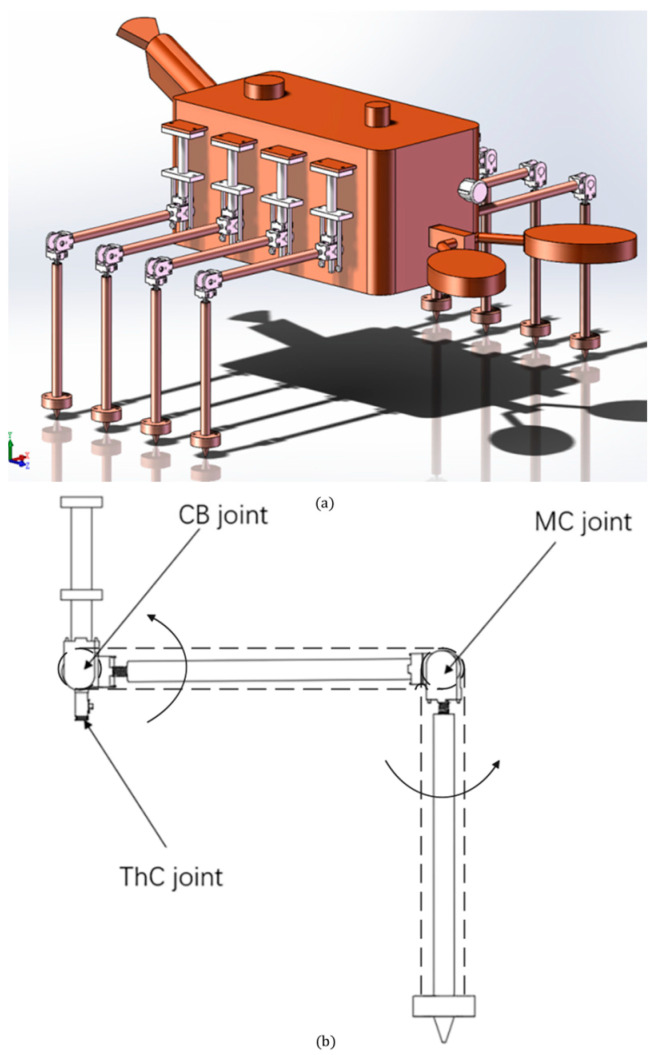
(**a**) Overall view of the machine lobster. (**b**) Structural view of the legs of the machine lobster (the dashed line in the figure represents SMA).

Inspired by lobsters, we have designed a biomimetic robotic lobster to cope with complex underground environments. In this study, the structural configuration of the machine lobster’s legs is illustrated in Figure 1.

Referring to the legs of lobsters, this study simplifies their legs into three degrees of freedom. The robotic structure under discussion comprises three primary joints: the basal joint (CB joint), the thoracic axis joint (ThC joint), and the carpal-foot joint (MC joint). The basal joint (CB joint) facilitates the elevation and depression of the legs within a horizontal plane. The thoracic axis joint (ThC joint) is responsible for the overall rotational movement of the leg structure in the axial direction. The carpometacarpal joint (MC joint) enables the contraction and extension of the foot, thereby influencing the foot–leg angle. During the forward and backward gait, the swing phase involves a rhythmic coordination of vertical rotation at the thoracic axis joints, elevation at the basal joints, and extension at the carpometacarpal joints to achieve the swing movement. In the support phase, the basal and carpometacarpal joints are locked, while the thoracoaxial joints rotate to facilitate the support movement. In the horizontal gait, the thoracic-axial joints are locked, allowing the basal joints and carpal-foot joints to work in concert to complete the movement. The overall design of the machine lobster is inspired by the anatomical structure of a real lobster, which typically consists of a head, thorax, abdomen, pectoral appendages (including chelae), gastropods, and a tail. The robotic version has been simplified to meet specific functional requirements; it omits the head, retains eight legs, includes one pincer, and features a simplified fan-shaped tail structure. In summary, the machine lobster designed in this study comprises a torso and legs, with the torso consisting of a box, pincers, and a tail, as illustrated in Figure 1b. The torso of the machine lobster serves as the primary control center, from which all control signals are transmitted. The eight legs are symmetrically and evenly affixed to both sides of the chassis, as illustrated in Figure 1b. Additionally, the pincers and tail of the machine lobster are designed to be removable and are secured to the front and rear baffles of the chassis using fasteners. The chassis is a critical component of the torso, as it houses the servos responsible for driving the movements of the leg joints, pincers, and tail, as well as the microcontroller that governs all servo operations. It is essential that these components are arranged as evenly as possible within the chassis to ensure that the center of mass of the entire machine lobster aligns with its geometric center, thereby maximizing the stability of its gait. The muscle actuator of the machine lobster functions as the physical module for specific motion control, exhibiting properties akin to biological muscles. In this study, shape memory alloys (SMAs) were selected as the muscle actuators. These alloys demonstrate a shape memory effect, characterized by the transformation from martensite to austenite.

**Figure 2:**
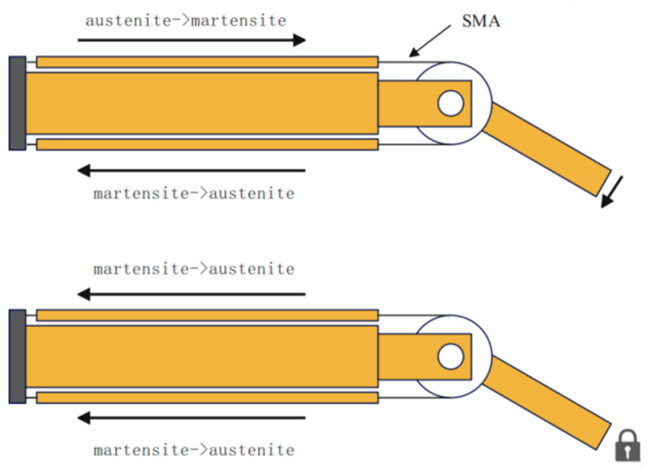
Schematic of joint control of SMAs.

This transformation results in shape contraction, enabling the shape memory metal to facilitate motion control [26]. The transition from martensite to austenite can be regulated through pulse-width modulation (PWM), wherein the duty cycle is adjusted to control joint movement. The specific control mechanism is depicted in Figure 2, which illustrates the placement of the memory metal on both sides of the joint, allowing for coordinated control of the joint’s motion through the varying transformation states of each side (one side relaxing while the other contracts). When joint locking is necessary, both sides of the memory metal collaborate to transition from martensite to austenite, resulting in simultaneous contraction at both ends to ensure effective joint locking [27]. The implementation of shape memory alloys (SMAs) can enhance the stability and flexibility of robotic lobsters when operated remotely in intricate subterranean environments.

### 2.2. Mathematical Modeling of a Machine Lobster

**Figure 3:**
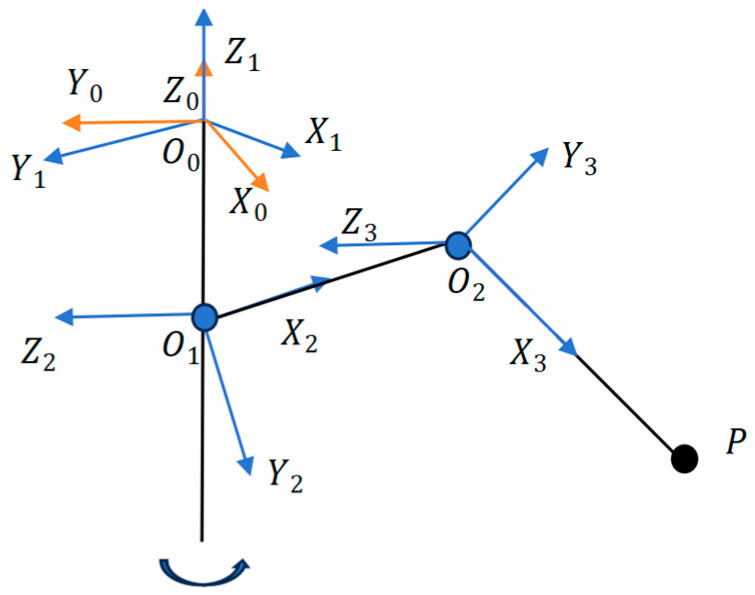
DH coordinate system diagram of the leg structure.

Analyzing the motion of the machine lobster first requires establishing positive kinematics according to the leg structure (Figure 1) of the physical modeling. First, we established the DH coordinate system based on the physical model, as shown in Figure 3.

We set the leg structure to the box link point as the base point $O_{0}$. We established a base coordinate system ($X_{0}Y_{0}Z_{0}$) at this point and established the $X_{1}Y_{1}Z_{1}$ coordinate system of the leg at the same point, according to the structure of the leg in order to establish the coordinate system. We set $O_{0}O_{1}=d_{1},O_{1}O_{2}=L_{1},O_{2}P=L_{2}$. Based on the coordinate system and parameters, the DH table of the leg structure can be obtained, i.e., Table 1.

According to the DH table of the leg structure with robotics, the state-transfer matrix Tii−1 can be written as Equation (1). In the equation, c represents the cosine function and s represents the sine function.


Tii−1=Transxai−1Rotxαi−1TranszdiRotzθi,

(1) Tii−1=cθi−sθi0ai−1sθicαi−1cθicαi−1−sαi−1−sαi−1disθisαi−1cθisαi−1cαi−1cαi−1di0001,

Substituting the parameters in the DH table into Equation (1) yields the state-transfer matrix T10, T21, T32:


T10=cosθ1−sinθ100sinθ1cosθ10000100001,

T21=cosθ2−sinθ20000−1−d1sinθ2cosθ2000001,

T32=cosθ3−sinθ30L1sinθ3cosθ30000100001,

According to the nature of state transfer matrices in robotics, T30 can be obtained using Equation (2). The rotation matrix that defines the relationship of attitude transformation is presented in the equation. Additionally, the position matrix $P={\left[p_{x},p_{y},p_{z}\right]}^{T},O=\left[0,0,0\right],I=1$ characterizes the relationship of positional changes.


(2) T30 = T10 × T21 × T32 = RPOI=nxoxaxpxnyoyaypynzozazpz0001,

In the equation:


$$n_{x}=c\theta_{1}c\theta_{2}c\theta_{3}-c\theta_{1}s\theta_{2}s\theta_{3},$$

$$n_{y}=c\theta_{2}c\theta_{3}s\theta_{1}-s\theta_{1}s\theta_{2}s\theta_{3},$$

$$n_{z}=c\theta_{2}s\theta_{3}+s\theta_{2}c\theta_{3},$$

$$o_{x}=-c\theta_{1}c\theta_{2}s\theta_{3}-c\theta_{1}s\theta_{2}c\theta_{3},$$

$$o_{y}=-c\theta_{2}s\theta_{3}s\theta_{1}-s\theta_{1}s\theta_{2}c\theta_{3},$$

$$o_{z}=c\theta_{2}c\theta_{3}-s\theta_{2}s\theta_{3},$$

$$a_{x}=s\theta_{1},a_{y}=-c\theta_{1},a_{z}=0,$$

$$p_{x}=d_{1}s\theta_{1}+a_{1}c\theta_{1}c\theta_{2},p_{y}=a_{1}s\theta_{1}c\theta_{2}-d_{1}c\theta_{1},p_{z}=a_{1}s\theta_{2},$$

We set Tp3=L2,0,0,1T. Then, the coordinates $P_{c}$ of the foot end P relative to the base coordinate system $X_{0}Y_{0}Z_{0}$ can be introduced by Equation (3).


(3) T30 × Tp3 = [Pc , 1],

$$P_{c}=[L_{2}n_{x}+p_{x}\;,\;L_{2}n_{y}+p_{y}\;,\;L_{2}n_{z}+p_{z}],$$

Robot inverse kinematics is the inverse solution of the angles ($\theta_{1},\theta_{2},\theta_{3}$) of joints with known coordinates of the P-point $\left[P_{x},P_{y},P_{z}\right]$. The system of equations can be solved by the $P_{c}$ obtained from the positive kinematics or directly by the geometric method. Because the geometry of the leg structure is very simple, in this study, the system of Equation (4) is obtained directly through the geometric method, where the projected length of the leg is set to R when viewed from the top of the $Z_{0}$-axis.


(4) $$\left\{\begin{array}{c} P_{x}=R\cos\theta_{1}\\ P_{y}=R\sin\theta_{1}\\ P_{z}=-d_{1}+l_{1}\sin\theta_{2}-l_{2}\sin\left(\theta_{3}-\theta_{2}\right)\\ R=l_{1}\cos\theta_{2}+l_{2}\cos\left(\theta_{3}-\theta_{2}\right) \end{array}\right.,$$

Solving the equation system:


(5) $$\theta_{1}=\tan^{-1}\left(\frac{P_{y}}{P_{x}})+k\pi \;\;(\theta_{1}\in (-\frac{\pi }{4},\frac{\pi }{4})∴k=0\right),$$

(6) $$\theta_{2}=\sin^{-1}(\frac{C_{1}+l_{2}\sin\left(\sin^{-1}\left(C_{3}\right)+\tan^{-1}(\frac{C_{2}}{C_{1}})+\pi \right)}{l_{1}}),$$

(7) $$\theta_{3}=\sin^{-1}(\frac{C_{1}+l_{2}\sin\left(\sin^{-1}\left(C_{3}\right)+\tan^{-1}\left(\frac{C_{2}}{C_{1}}\right)\right)}{l_{1}})+\sin^{-1}\left(C_{3}\right)+\tan^{-1}(\frac{C_{2}}{C_{1}})+\pi ,$$

$$C_{1}=P_{z}+d_{1},C_{2}=\frac{P_{x}}{\cos\left(\tan^{-1}\left(\frac{P_{y}}{P_{x}}\right)\right)},C_{3}=\frac{{{{l_{1}}^{2}-(C}_{2}}^{2}+{l_{2}}^{2})}{2l_{2}\sqrt{{C_{1}}^{2}+{C_{2}}^{2}}}.$$

### 2.3. High Order Fully Driven Dynamic Modeling

To cope with complex underground dynamic environments such as sudden changes in loose rock layers and impacts from unstructured terrain, this paper conducted a high-order fully driven dynamic modeling of robotic lobsters to achieve precise control.

The robotic lobster single leg consists of three rotating joints (ThC joint, CB joint, MC joint), corresponding to generalized coordinates $q={\left[\theta_{1},\theta_{2},\theta_{3}\right]}^{T}$. To achieve high-order full drive of robotic crayfish, it is necessary to conduct rigid body kinematic modeling. The modeling of rigid body kinematics is mainly divided into the Newton Euler method and Lagrange method. The Newton Euler method analyzes from the perspective of mechanics, with more detailed calculations and larger computational complexity. The Lagrangian method analyzes from the perspective of energy and has relatively simple calculations, so this article chose the Lagrangian method for kinematic modeling.

The Lagrangian function is expressed as Equation (8), which is a function that interprets motion from an energy perspective. L is the Lagrangian (unit J), T is the kinetic energy (unit J), and V is the potential energy (unit J).

(8) L = T − V,

Further derivation can lead to another form of the Lagrange equation, where τ is the generalized joint output torque (N·mm); q is the Joint angle (rad); $\dot{q}$ is the Joint angular velocity (rad/s); and L is the Lagrangian (J).

(9) $$\tau =\frac{d}{dt}\frac{\partial L}{\partial \dot{q}}-\frac{\partial L}{\partial q},$$

Equation (9) can be used to calculate the driving torque required for each joint of the robotic lobster. Due to the two motion states of the robotic lobster, namely, the support phase and the swing phase, which require different torques, they need to be discussed separately.

The kinetic energy of the swinging phase of the robotic lobster is divided into two parts: translational kinetic energy and rotational kinetic energy. The legs of the robotic lobster are composed of three segments, each of which is a homogeneous connecting rod. The kinetic energy analysis of each connecting rod will be conducted based on the model of the robotic lobster.

The first connecting rod is responsible for vertical rotation and only has rotational kinetic energy. In Equation (10), $I_{1}$ is the moment $\dot{\theta_{1}}$ of inertia of the first connecting rod and is the rotational speed of the first connecting rod around the ThC joint.

(10) $$T_{1}=\frac{1}{2}I_{1}{{\dot{\theta }}_{1}}^{2},$$

The kinetic energy of the second connecting rod is divided into two parts, namely, the kinetic energy rotating around ThC and the kinetic energy rotating around the CB joint, which are perpendicular to each other. The kinetic energy of this connecting rod can be directly integrated to obtain. $m_{2}$ is the mass of the second connecting rod, $L_{1}$ is its length, and the ratio of the two is the linear density. r is the distance between the mass element and the origin.

$$\begin{array}{l} T_{2}=\frac{m_{2}}{2L_{1}}\int_{0}^{L_{1}}\left({{\dot{\theta }}_{1}}^{2}r^{2}{cos}^{2}\theta_{2}+{{\dot{\theta }}_{2}}^{2}r^{2}\right)dr\;\\ =\frac{m_{2}L_{1}^{2}}{6}\left({{\dot{\theta }}_{1}}^{2}{cos}^{2}{{\dot{\theta }}_{2}}^{2}+{{\dot{\theta }}_{2}}^{2}\right), \end{array}$$

The kinetic energy of the third connecting rod is divided into three parts: the kinetic energy of rotating around the ThC joint, the kinetic energy of rotating around the CB joint, and the kinetic energy of rotating around the MC joint. Due to its complexity, the velocity of rotating around the CB joint and the MC joint can be derived from the centroid coordinates, and the kinetic energy of rotating around the ThC joint can be calculated according to the kinetic energy formula of a homogeneous connecting rod rotating around the external axis of the connecting rod. The final kinetic energy is obtained as Equation (11).

(11) $$T_{3}=\frac{1}{2}m_{2}[{{\dot{\theta }}_{2}}^{2}{L_{1}}^{2}+\frac{{L_{2}}^{2}}{4}{\left({\dot{\theta }}_{3}-{\dot{\theta }}_{2}\right)}^{2}-{\dot{\theta }}_{2}L_{1}L_{2}\left({\dot{\theta }}_{3}-{\dot{\theta }}_{2}\right)\cos\theta_{3}]+\frac{1}{24}m_{2}{L_{2}}^{2}{\left({\dot{\theta }}_{3}-{\dot{\theta }}_{2}\right)}^{2}+\frac{1}{2}m_{3}{{\dot{\theta }}_{1}}^{2}[{L_{1}}^{2}{cos}^{2}\theta_{2}+\;L_{1}L_{2}cos\theta_{2}cos\left(\theta_{3}-\theta_{2}\right)+\frac{{L_{2}}^{2}}{3}],$$

Next, calculate the potential energy part and select the zero potential energy point at the connection between the first and second connecting rods.

$$V_{1}=\frac{1}{2}m_{1}gd_{1},$$

$$V_{2}=\frac{1}{2}m_{2}gL_{1}\sin\theta_{2},$$

$$V_{3}=m_{3}g\left[L_{1}\sin\theta_{2}-\frac{1}{2}\sin(\theta_{3}-\theta_{2})\right],$$

According to Equation (9), the torque of three joints can be calculated:

$$\tau_{1}=I_{1}{\ddot{\theta }}_{1}+\frac{m_{2}{L_{1}}^{2}}{3}({\ddot{\theta }}_{1}{cos}^{2}\theta_{2}-2{\dot{\theta }}_{1}{\dot{\theta }}_{2}sin\theta_{2}cos\theta_{2})+m_{3}{\ddot{\theta }}_{1}[{L_{1}}^{2}cos\theta_{2}\cos\left(\theta_{3}-\theta_{2}\right)+\frac{{L_{2}}^{2}}{3}]+\;m_{3}{\dot{\theta }}_{1}[-2{L_{1}}^{2}{\dot{\theta }}_{2}sin\theta_{2}cos\theta_{2}-L_{1}L_{2}{\dot{\theta }}_{2}sin\theta_{2}cos\left(\theta_{3}-\theta_{2}\right)+L_{1}L_{2}{\dot{\theta }}_{2}cos\theta_{2}sin\left(\theta_{3}-\theta_{2}\right)\left({\dot{\theta }}_{3}-{\dot{\theta }}_{2}\right)],$$

$$\tau_{2}=\frac{m_{2}{L_{1}}^{2}}{3}{\ddot{\theta }}_{2}+\frac{m_{2}}{2}[2{\ddot{\theta }}_{2}{L_{1}}^{2}-\frac{{L_{2}}^{2}}{2}\left({\dot{\theta }}_{3}-{\dot{\theta }}_{2}\right)-L_{1}L_{2}\cos\theta_{3}\left({\ddot{\theta }}_{3}-{\ddot{\theta }}_{2}\right)+L_{1}L_{2}{\dot{\theta }}_{3}sin\theta_{3}\left({\dot{\theta }}_{3}-{\dot{\theta }}_{2}\right)-\;{\ddot{\theta }}_{2}L_{1}L_{2}\cos\theta_{3}\left({\dot{\theta }}_{3}-1\right)+L_{1}L_{2}{\dot{\theta }}_{2}{\dot{\theta }}_{3}sin\theta_{3}\left({\dot{\theta }}_{3}-1\right)-{\dot{\theta }}_{2}{\ddot{\theta }}_{3}L_{1}L_{2}\cos\theta_{3}]-\frac{1}{12}m_{2}{L_{2}}^{2}\left({\ddot{\theta }}_{3}-{\ddot{\theta }}_{2}\right)+\;\frac{m_{2}{L_{1}}^{2}}{3}{{\dot{\theta }}_{1}}^{2}sin\theta_{2}cos\theta_{2}-\frac{1}{2}m_{3}{{\dot{\theta }}_{1}}^{2}[-{L_{1}}^{2}sin\theta_{2}cos\theta_{2}-L_{1}L_{2}sin\theta_{2}cos\left(\theta_{3}-\theta_{2}\right)+L_{1}L_{2}cos\theta_{2}sin\left(\theta_{3}-\theta_{2}\right)]+\;\frac{1}{2}m_{2}gL_{1}\cos\theta_{2}+m_{3}gL_{1}\cos\theta_{2}+\frac{1}{2}m_{3}gL_{2}cos\left(\theta_{3}-\theta_{2}\right),$$

$$\tau_{3}=\frac{m_{2}}{2}[2{\dot{\theta }}_{2}{\ddot{\theta }}_{2}{L_{1}}^{2}+\frac{{L_{2}}^{2}}{2}\left({\ddot{\theta }}_{3}-{\ddot{\theta }}_{2}\right)-L_{1}L_{2}{\ddot{\theta }}_{2}\left(1-{\dot{\theta }}_{2}\right)\cos\theta_{3}+L_{1}L_{2}{\dot{\theta }}_{2}{\ddot{\theta }}_{2}\cos\theta_{3}+L_{1}L_{2}{\dot{\theta }}_{2}{\dot{\theta }}_{3}\left(1-{\dot{\theta }}_{2}\right)sin\theta_{3}]+\;\frac{1}{12}m_{2}{L_{2}}^{2}\left({\ddot{\theta }}_{3}-{\ddot{\theta }}_{2}\right)-\frac{m_{2}}{2}L_{1}L_{2}{\dot{\theta }}_{2}\left({\dot{\theta }}_{3}-{\dot{\theta }}_{2}\right)sin\theta_{3}+\frac{1}{2}m_{3}{{\dot{\theta }}_{1}}^{2}L_{1}L_{2}cos\theta_{2}sin\left(\theta_{3}-\theta_{2}\right)+\frac{1}{2}m_{3}gL_{2}cos\left(\theta_{3}-\theta_{2}\right),$$

For the derivation of the dynamics during the stance phase, according to the research by Grzelczyk et al., the foot impact force is approximately one order of magnitude lower than the reaction force exerted on the foot sole [17]. Therefore, this paper neglects the foot impact force and considers only gravitational force and foot-end friction force for model simplification. The force on the foot end is as shown in Equation (12). The gait of an eight-legged robot is similar to the Tetrapod Gait, with 4–5 legs in the supporting phase at any given time, bearing the weight and inertial force of the body. The average value for this article is 4.5. μ is the coefficient of friction.

(12) $$F_{e}={\left[F_{x},F_{y},F_{z}\right]}^{T}={\left[\mu \frac{G}{4.5},0,-\frac{G}{4.5}\right]}^{T},$$

The formula for calculating the $F_{e}$ torque of each joint is Equation (13). Since the swing phase torque has already been calculated, it is only necessary to calculate the transpose of the Jacobian matrix $J^{T}$ and multiply it with the force vector at the foot end to obtain the torque of each joint.

(13) $$\tau_{i}=\frac{d}{dt}\frac{\partial L}{\partial {\dot{\theta }}_{i}}-\frac{\partial L}{\partial \theta_{i}}+J^{T}F_{e},$$

$$J^{T}=\left[\begin{array}{ccc} a_{11} & a_{12} & a_{13}\\ a_{21} & a_{22} & a_{23}\\ a_{31} & a_{32} & a_{33} \end{array}\right],$$

$$a_{11}=-[L_{1}cos\theta_{2}+L_{2}cos\left(\theta_{3}-\theta_{2}\right)]sin\theta_{1},$$

$$a_{12}=[L_{1}cos\theta_{2}+L_{2}cos\left(\theta_{3}-\theta_{2}\right)]cos\theta_{1},$$

$$a_{13}=0,$$

$$a_{21}=[-L_{1}sin\theta_{2}+L_{2}sin\left(\theta_{3}-\theta_{2}\right)]cos\theta_{1},$$

$$a_{22}=[-L_{1}sin\theta_{2}+L_{2}sin\left(\theta_{3}-\theta_{2}\right)]sin\theta_{1},$$

$$a_{23}=-d_{1}+l_{1}\cos\theta_{2}-l_{2}\cos\left(\theta_{3}-\theta_{2}\right),$$

$$a_{31}=-L_{2}cos\theta_{1}sin\left(\theta_{3}-\theta_{2}\right),$$

$$a_{32}=-L_{2}sin\theta_{1}sin\left(\theta_{3}-\theta_{2}\right),$$

$$a_{33}=-L_{2}cos\left(\theta_{3}-\theta_{2}\right).$$

The joint torque of the supporting phase is the $J^{T}F_{e}$ corresponding term added to the torque of the swinging phase.

### 2.4. Machine Lobster Gait Design

**Figure 4:**
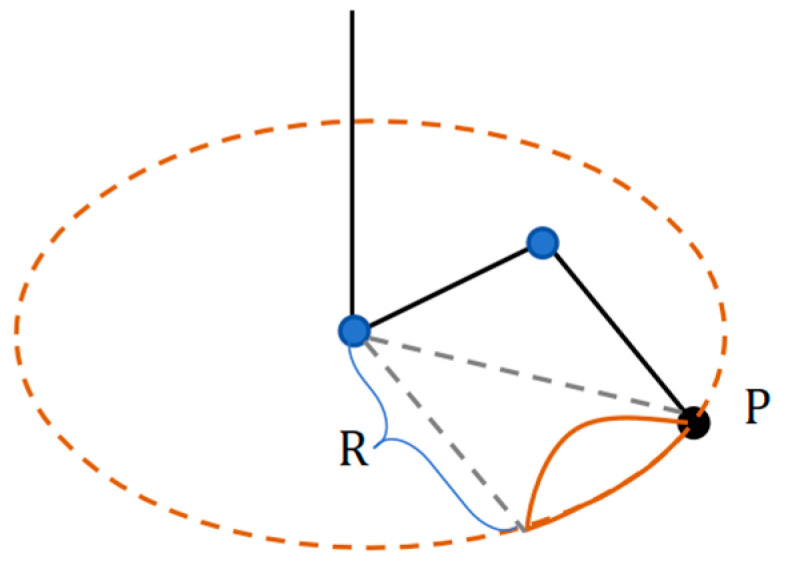
Schematic diagram of monopodial gait of machine lobster.

The design of the gait for the machine lobster commences with the development of the fundamental leg support and swing pattern. In the monopodial gait illustrated in Figure 4, the solid brown line represents the trajectory traced by the foot end, denoted as point P. The top view depicts the radius R corresponding to a segment of the arc. The equation governing this arc is labeled as $X^{2}+Y^{2}=R^{2}(Y\geq \sqrt{R^{2}-\frac{l^{2}}{4}})$. Additionally, the side view from the center of this arc to the center of the circle also forms an arc on the ground, represented by the equation $x^{2}+y^{2}=r^{2}(y\geq d)$. The common chord connecting these two arcs is designated as $l=2\sqrt{r^{2}-d^{2}}$, with its corresponding equation being $l=2\sqrt{r^{2}-d^{2}}$.

**Figure 5:**
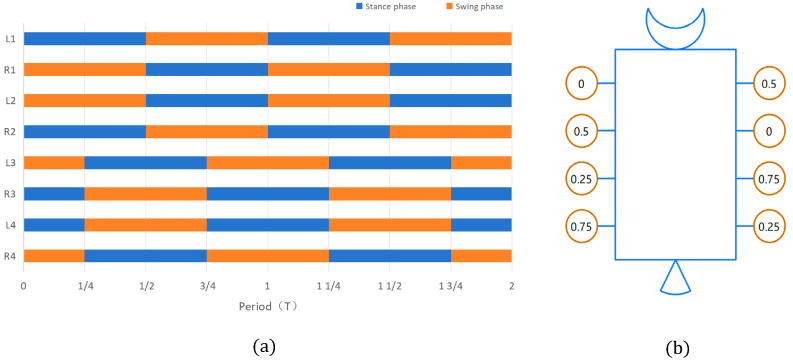
(**a**) Octopod gait diagram. (**b**) Octopod gait timing diagram of the machine lobster.

The gait of octopod robotic systems, such as the machine lobster, exhibits characteristics analogous to those observed in quadrupedal and hexapod robots [28,29]. This gait’s walking efficiency does not have an advantage on flat terrain, but it is suitable for straight-line walking in complex environments. In this context, L denotes the left foot of the machine lobster, while R represents the right foot. The gait pattern of the initial four limbs (i.e., L1, R1, L2, R2) closely resembles the trot gait typical of quadrupedal robots, as illustrated in Figure 5. Specifically, L1 is positioned in opposition to the support swing phase of R1, and L2 is placed in opposition to the support swing phase of R2. Furthermore, L1 and R2 share the same motion state, as do L2 and R1. Notably, the motion state of the final four limbs lags behind that of the first four limbs by a quarter of the cycle, maintaining a one-to-one correspondence. To exemplify this, the gait of the eight limbs over two cycles is depicted in Figure 5a.

A timing diagram with a period of 1.0 is presented in Figure 5b. These two diagrams complement one another in illustrating the specific instance of the octopod gait of the machine lobster.

### 2.5. Simulation Validation of the Mathematical Parameters of the Machine Lobster

To ensure the accuracy and stability of joint movements within complex underground geological environments, as well as the precision and feasibility of each parameter and derivation in physical and mathematical modeling, this study employed MATLAB simulations to analyze the relevant formulas of $\theta_{1},\theta_{2},\theta_{3}$. The objective was to observe the impact of varying the coordinates $\left[P_{x},P_{y},P_{z}\right]$ of the foot end position of the machine lobster on the rotational angles of each joint. Ultimately, this analysis aimed to determine the parameters of the physical model of the machine lobster by examining the feasible intervals obtained, as well as to validate the reasonableness of the formulas derived through mathematical modeling. Additionally, it can ensure that the machine lobster maintains optimal joint motion posture during remote control operations.

**Figure 6:**
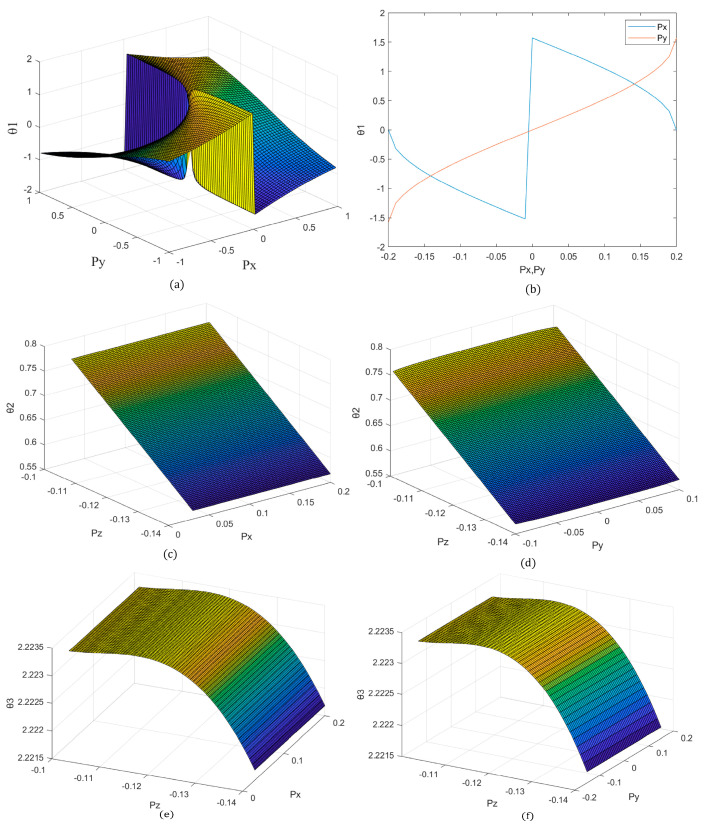
(**a**) Diagram of the corner versus end-of-foot position. (**b**) Diagram of the corner versus end-of-foot position after entering monopodial gait. (**c**) Relationship of the corner versus end-of-foot position after entering monopodial gait. (**d**) Relationship of the corner versus end-of-foot position after entering monopodial gait. (**e**) Relationship between the corner and end-of-foot position after entering monopodial gait. (**f**) Relationship between the corner and foot-end position after entering monopodial gait.

From Figure 6a, it can be observed that there exists a substantially feasible domain for the foot end position $P_{x},P_{y}$ when condition $\theta_{1}\in (-\frac{\pi }{4},\frac{\pi }{4})$ must be satisfied, for which $\theta_{1}$ and $P_{z}$ are unrelated; therefore, the mathematical modeling in this study is deemed reasonable. In the actual lobster movement, the coordinates $\left[P_{x},P_{y},P_{z}\right]$ of the foot end position are not independent but rather interconnected through the monopodial gait (i.e., constructed in II.C in the single-footed gait). The relationship between the angles of rotation $\theta_{1}$ and $P_{x},P_{y}$ in gait can be reduced to Figure 6b. This form of reduction can similarly yield separate feasible intervals for $P_{x},P_{y}$ through the relationship $P_{x},P_{y}$, which can be derived from the feasible intervals of the other parameter.

From Equations (6) and (7), it is evident that $\theta_{2}$ and $\theta_{3}$ are related to all three parameters of $\left[P_{x},P_{y},P_{z}\right]$ without considering the gait; consequently, it is not feasible to observe the simulation. In the case of monopodial gait, it is possible to analyze the relationship between $\theta_{2},\theta_{3}$, and $\left[P_{x},P_{y},P_{z}\right]$ by reducing the dimensions to three. The relationship between $\theta_{2}(rad)$ and $P_{x},P_{y},P_{z}$ is illustrated in Figure 6c,d: as $\left|P_{z}\right|$ decreases, $\theta_{2}$ increases. The value of $\theta_{2}$ is independent of $P_{x},P_{y}$. This observation aligns with the actual situation and is not discernible in the simulation. This correlation is consistent with the physical scenario that when $\left|P_{z}\right|$ decreases, the P point increases, the basal joint (CB joint) rises, and the angle increases. Upon entering a monopodial gait, $P_{x},P_{y}$ alone cannot influence the value of $\theta_{2}$; however, the radius of the circle they form, R, does. This indicates that the relationship between $\theta_{2}$ and $\left[P_{x},P_{y},P_{z}\right]$ is consistent with the actual situation and is accurate and feasible.

Similarly, the relationship between $\theta_{3}(rad)$ and $P_{x},P_{y},P_{z}$, as depicted in Figure 6e,f demonstrates that when $\left|P_{z}\right|$ decreases, $\theta_{3}$ initially increases and then decreases slightly, again independent of $P_{x},P_{y}$. This corresponds to the actual situation: when $\left|P_{z}\right|$ decreases, the P point increases, and in the case of unchanged R in monopodial gait, in the intermediate positions between the two limit positions of $\theta_{3}=\pi$, the angle of the carpal-foot joint (MC joint) exhibits an initial increase followed by a decrease (analogous to the crank linkage mechanism). In conclusion, the formula derived through mathematical modeling is reasonable and feasible. The final determination of the most reasonable and feasible physical parameters for each component of the machine lobster is presented in Table 2. $L_{B},W_{B},H_{B}$ in the table represents the length, width, and height of the machine lobster box, whereas $d_{1},L_{1},L_{2}$ represents the parameters for the legs of the machine lobster.

## 3. Machine Lobster Modeling

**Figure 7:**
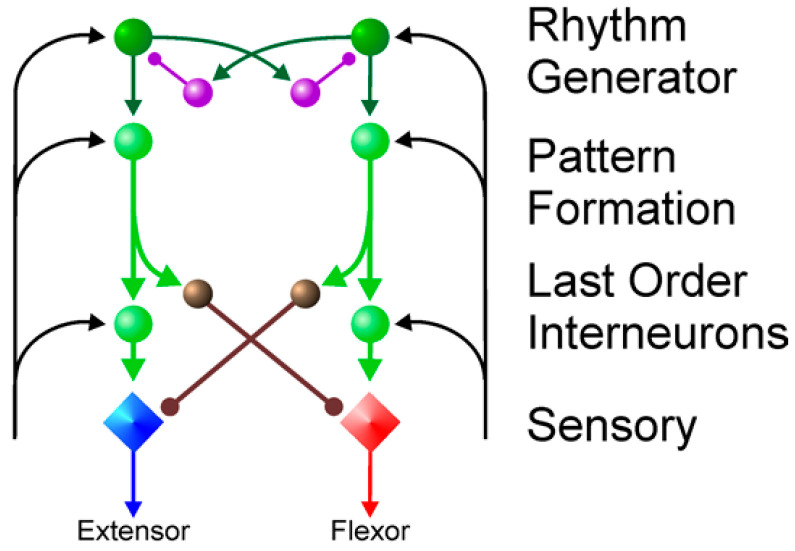
Multi-layer CPG network proposed by McCrea, D. et al. [30].

McCrea et al. [30] proposed a multilayered central pattern generator (CPG) network that facilitates multifaceted multi-degree-of-freedom gait control. As depicted in Figure 7, this enhanced flexibility stems from the integration of a pattern formation layer, which serves as an intermediary between the upper and lower levels, receiving rhythmic inputs from the generator to modulate extension and flexion movements through intricate neuron interconnections. This configuration enhances the control adaptability and introduces a more articulated structural rationale. Given the intricate nature of the octopod lobster-inspired robot, this study adopted a multilayered CPG neural network architecture for optimal performance.

### 3.1. Selection of Rhythm Generators

The mathematical formulation of the Kuramoto nonlinear oscillator, chosen for this investigation, is presented as follows:

$${\dot{\theta }}_{i}=w_{i}+\sum_{j=1}^{N}\;K_{ij}sin\left(\theta_{j}-\theta_{i}\right)\;\;\;\;i,j\epsilon N^{*},i\neq j,$$

The phase of the i oscillator in ${\dot{\theta }}_{i}$ is determined, where $w_{i}$ denotes the oscillator’s intrinsic frequency and $K_{ij}$ represents the coupling strength between oscillator i and oscillator j. Synchronization occurs when the coupling strength between these oscillators attains a critical threshold, aligning their phases. Nonetheless, the original mathematical model lacks the capacity to generate rhythmic signals that incorporate phase differences, thereby precluding the realization of rhythmic control with phase-dependent limb joint coordination. To address this limitation, advancements have led to the formulation of the Kuramoto model, which incorporates the frustration effect [31] and enhances its versatility:

(14) $${\dot{\theta }}_{i}=w_{i}+\sum_{j=1}^{N}K_{ij}\;sin\left(\theta_{j}-\theta_{i}-\varphi_{ij}\right)\;\;\;\;i,j\epsilon N^{*},i\neq j,$$

The introduction of frustrated phase $\varphi_{ij}$ enables the phase difference between oscillators i and j to converge to $\varphi_{ij}$ when the coupling strength is optimally tuned, thereby facilitating the generation of rhythmic signals with distinct and controlled phase differences. This approach offers enhanced flexibility in neural network construction and clarity in control logic, motivating the adoption of this oscillator for rhythmic output in the present study.

The mathematical model of the interneuron, which is grounded in the Kuramoto model and incorporates the frustration effect, is formulated in Equation (15) for a specific phase-locking scenario, as shown in the following equation:

(15) $${\dot{\theta }}_{pi}={2\pi w}_{pi}+\sum_{j=1}^{N}K_{pij}\;sin\left(\theta_{pj}-\theta_{pi}-\varphi_{pij}\right)\;\;\;\;i,j\epsilon N^{*},i\neq j,$$

**Figure 8:**
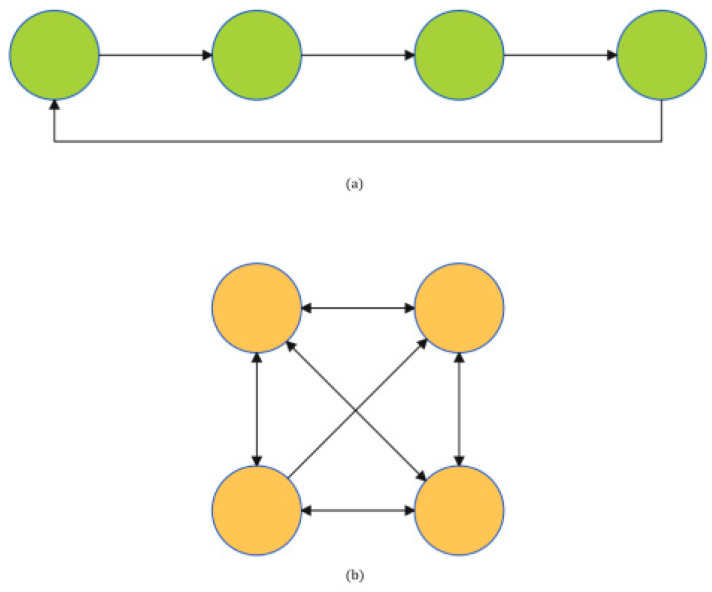
(**a**) Chain structure of interneurons. (**b**) Mesh structure of interneurons.

The efficacy of the CPG control method in achieving intricate motion control stems from its ability to construct neural networks with intricate topologies using intermediary neurons. Subsequently, these networks govern the lower motor neurons, ultimately facilitating the realization of desired control effects. Currently, neural network architectures primarily consist of chain and grid structures, as illustrated in Figure 8.

In practical control applications, chain-structured neural networks are predominantly employed in serial-logic-based control processes, as exemplified by snake-like robots [32], where joints arranged sequentially are subjected to rhythmic control in tandem, enabling serpentine locomotion through phase differences.

**Figure 9:**
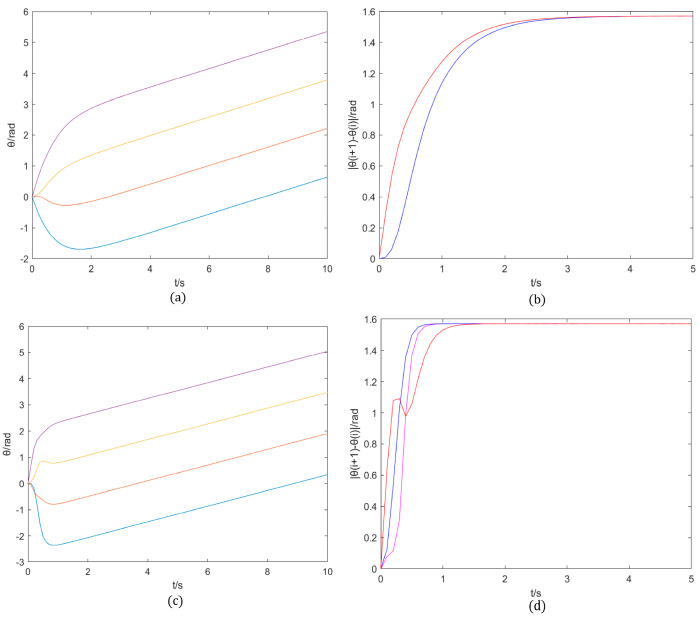
Difference between (**a**,**b**) chain structure of interneurons and (**c**,**d**) mesh structure of interneurons.

Conversely, mesh-structured neural networks are primarily utilized in parallel-logic-driven control scenarios, as observed in quadrupedal robots [33], where independent gait patterns across four limbs are orchestrated by parallel rhythms, maintaining stable phase disparities. A comparative analysis of the two architectures can be performed by developing their respective neural networks that incorporate the rhythm generator of the Kuramoto model, while also considering the effects of frustration, as depicted in Figure 9.

After multiple experimental measurements, the frequency $w_{i}$ must remain consistent within the same network, the coupling strength $K_{ij}$ should be moderate and appropriate (to ensure fast convergence and prevent overshooting), and the frustration phase $\varphi_{ij}$ should be determined based on the required phase relationship. Given $w_{i}=0.3$, $K_{ij}=3$, $\varphi_{ij}=\left|i-j\right|\left|\frac{\pi }{2}\right|$, a comparative analysis of the two networks converging to a specific value indicates that the chain structure demonstrates a significantly slower rate of convergence compared to the mesh structure. This difference can be attributed to the sequential reception of rhythms by intermediate neurons within the chain structure, which results in a delayed stabilization of phase differences. Conversely, the mesh structure promotes rapid convergence, a critical factor for the synchronized control of multiple rhythms. Consequently, the machine lobster, operating under remote sensing control, necessitates the implementation of parallel logic to achieve rapid and synchronized control. Additionally, it emphasizes the selection of selecting neural networks distinguished by grid structures.

In essence, a motor neuron constitutes a robust dynamical control system, where the prevailing framework of contemporary control theory posits that the most comprehensive depiction of its state space is encapsulated by the following model:

$$\left\{\begin{array}{c} \dot{x}=f(x,u,t)\\ y=g(x,u,t) \end{array}\right.,$$

In the context of the kinetic system, particularly pertaining to the motor neuron, x represents the state variable, specifically designating the phase θ of the output emanating from the intermediate neuronal entity. State variable u represents the phase of the output from the intermediate neurons within the motor neuron kinetic system, whereas t and y denote the input variable and time, respectively. Specifically, in motor neuron systems, f(x,u,t) acts as the output variable governing the joint and muscle control signals, thereby reflecting the state of the system. g(x,u,t) functions as the output function. To enable precise robot motion control, this generalized dynamical system must be tailored to a specific and suitable form that adheres to certain prerequisites. It must possess the capability to process interneuronal signals and output arbitrary rhythmic periodic signals, which are pivotal for the motor neuron orchestration of intricate robotic movements. Additionally, it must maintain time invariance and stability, which are essential for seamless robotic motion control, ensuring continuous and non-abrupt alterations in the actuation commands $y,\dot{y},\ddot{y}$. The system’s output should exhibit single coupling with rhythm, where phase variations solely mirror rhythmic changes, unaffected by amplitude, frequency, or overall signal shape.

Auke Jan Ijspeert et al. [34,35] introduced a dynamical system that meets these criteria, adopting a modulated damped spring model incorporating a nonlinear term, as described by the following mathematical formulation:

(16) $$\left\{\begin{array}{c} \tau \dot{z}=\alpha_{z}\left(\beta_{z}(g-y)-z\right)+f\\ \tau \dot{y}=z \end{array}\right.,$$

The time constant is denoted by τ, whereas $\alpha_{z}$ and $\beta_{z}$ are positive constants constrained by the condition $\alpha_{z}=4\beta_{z}$, ensuring strict convergence of the system (classified as proximity-damped). The state variables of the system are z and y, and f serves as the forcing term for extracting the desired signal. Notably, the system possesses a unique point attractor at (z,y)=(0,g), implying the convergence of the system state to (0,g) under the condition f=0. The structure of the forcing term f is contingent upon the motor neuron’s signal output; for periodic rhythm-based outputs, Auke Jan Ijspeert et al. [34,35] proposed a specific form of f, which is detailed as follows:

$$f_{\mathrm{target}}=\tau^{2}{\ddot{y}}_{\mathrm{demo}}-\alpha_{z}\left(\beta_{z}\left(g-y_{\mathrm{demo}}\right)-\tau {\dot{y}}_{\mathrm{demo}}\right),$$

Given a known output $y_{\mathrm{demo}}$, it is incorporated into Equation (16) to derive the desired form of the target forcing term $f_{\mathrm{target}}$. Subsequently, to account for the frustration effect, the Kuramoto model-based input to the intermediate neuron was derived by substituting Equation (14) for $y_{\mathrm{demo}}$. Finally, output $y_{\mathrm{demo}}$ is recalculated by substituting Equation (16) back into its corresponding position.

$$f_{\mathrm{target}}=\tau^{2}{\dot{\theta }}^{2}{\ddot{y}}_{demo}\left(\theta \right)+\tau \alpha_{z}\dot{\theta }{\dot{y}}_{demo}\left(\theta \right)+\alpha_{z}\beta_{z}y_{demo}\left(\theta \right)-\alpha_{z}\beta_{z}g,$$

The culmination of the motor neuron power system formulation involves substituting the derived $f_{\mathrm{target}}$ into Equation (10).

$$\left\{\begin{array}{l} \tau \dot{z}=\tau^{2}{\dot{\theta }}^{2}{\ddot{y}}_{demo}\left(\theta \right)+\tau \alpha_{z}\left(\dot{\theta }{\dot{y}}_{demo}\left(\theta \right)-\dot{y}\right)+\alpha_{z}\beta_{z}\left(y_{demo}\left(\theta \right)-y\right)\\ \tau \dot{y}=z \end{array}\right.,$$

This study employed a kinetic model tailored for motor neurons capable of integrating target signal inputs with the rhythmic patterns of intermediate neurons, thereby generating a stable and targeted output signal. This guarantees that the machine lobster is capable of executing tasks reliably in subterranean and mining environments.

### 3.2. CPG Control Methods in Machine Lobster

**Figure 10:**
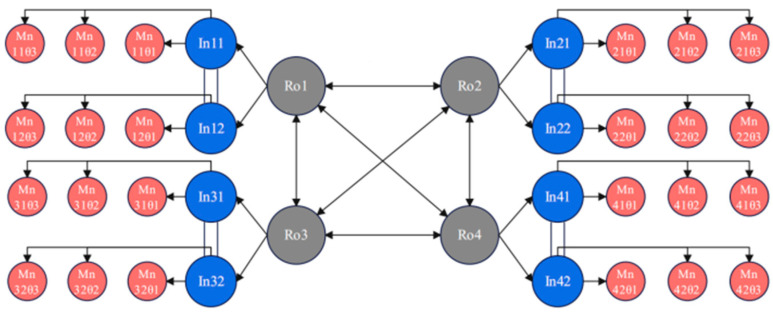
Schematic diagram of CPG control structure for machine lobsters.

The presence of four distinct temporal sequences within the octopod system of the machine lobster, as illustrated in Figure 5, necessitates the synchronization of these four rhythms to regulate their operations effectively. Figure 10 demonstrates the implementation of CPG control within this octopod machine lobster. These four rhythms utilize a mesh-like configuration to enhance response times, with each rhythm independently managing a pair of identical intermediate neurons, a design choice informed by the uniformity of gait patterns. These intermediate neurons exert control over the trio of motor neurons designated for each leg, thereby determining the angular positions of the three joints within the leg. This coordinated control mechanism successfully achieves the movement objectives of the machine lobster.

## 4. Simulation and Analysis of the Machine Lobster CPG Network

### 4.1. Machine Lobster Octopod Front-Back Gait Simulation

**Figure 11:**
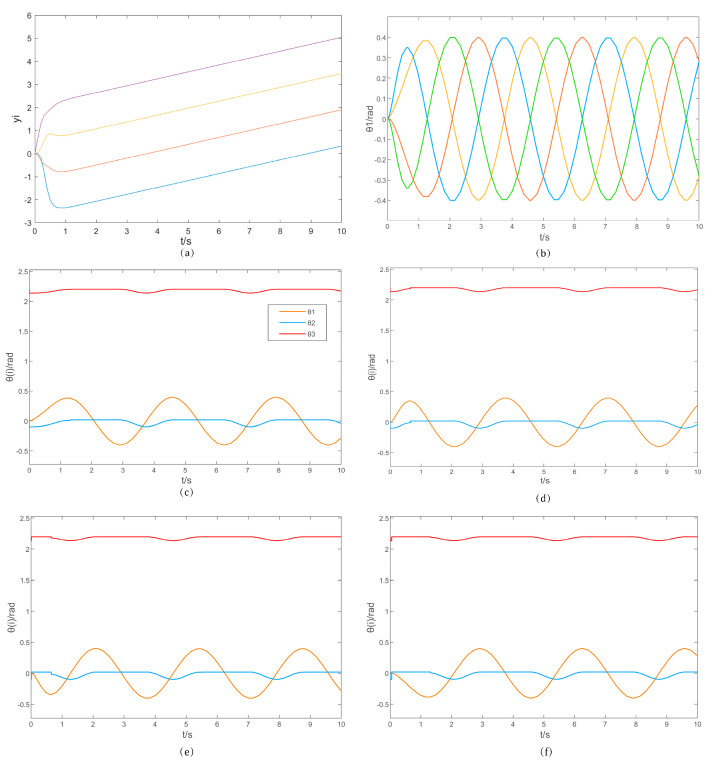
(**a**) Output of four rhythms. (**b**) Output of thoracic axis joint angles. (**c**–**f**) Variation of each joint angle under the control of the four rhythms according to the octopod gait.

The design of the anterior and posterior gait in II.D was implemented and simulated in Matlab/Simulink using ode45. The overall CPG neural network structure of the machine lobster is utilized as described in III.C. The distance between the ground and $X_{0}{OY}_{0}$ surface was assumed to be $l_{0}=0.22\;m$. The parameters R=0.2 m and R=0.2 m were established. The dimensional parameters of the machine lobster are presented in Table 2. Based on the determined parameters, the simulation model of the four rhythmic mesh structures was initially established, resulting in the simulation output depicted in Figure 11.

Drawing upon these four distinct rhythms, eight intermediary neural units were devised. By employing these neural intermediates alongside the formulated mathematical framework, the regulation of thoracic-axial $\theta_{1}$, basal $\theta_{2}$, and carpopedal $\theta_{3}$ joints was achieved. Specifically, the thoracic-axial joint $\theta_{1}$ was programmed to articulate in a sinusoidal, fluid motion, whereas the dynamics of the basal $\theta_{2}$ and carpopedal $\theta_{3}$ joints are governed by the inherent relationships encoded within the mathematical model. The resulting trajectory of the thoracic-axial joint angle $\theta_{1}$ is depicted in the accompanying figure. Furthermore, post-convergence, the four rhythms adhere rigorously to a stable sinusoidal pattern and adhering precisely the prescribed phase relationships.

The periodic variations in joint angles during forward and backward locomotion, governed by the four rhythmic controls, are illustrated in Figure 11c–f. Upon convergence, the joint angles evolved smoothly in harmony with the rhythms, fulfilling the stability criteria of the control system. Notably, the angles of all three joints remained within realistic and prescribed limits, with joint $\theta_{1}$ adhering strictly to the designated rhythm and pattern. During the swing phase, joints $\theta_{2}$ and $\theta_{3}$ exhibited dynamic changes commensurate with the swing, whereas they remained static during the support phase. The phase differences among the joints were stabilized by the rhythmic controls, tailored to the specific problem context, ensuring a coherent and realistic overall performance.

In conclusion, the simulation of the central pattern generator (CPG) network within the machine lobster illustrates the stability and viability of the CPG neural network architecture presented in this study. This network successfully generates realistic rhythmic and control signals, thereby enabling the execution of fundamental locomotion in the machine lobster.

### 4.2. Adams Simulation of Machine Lobster Motion

**Figure 12:**
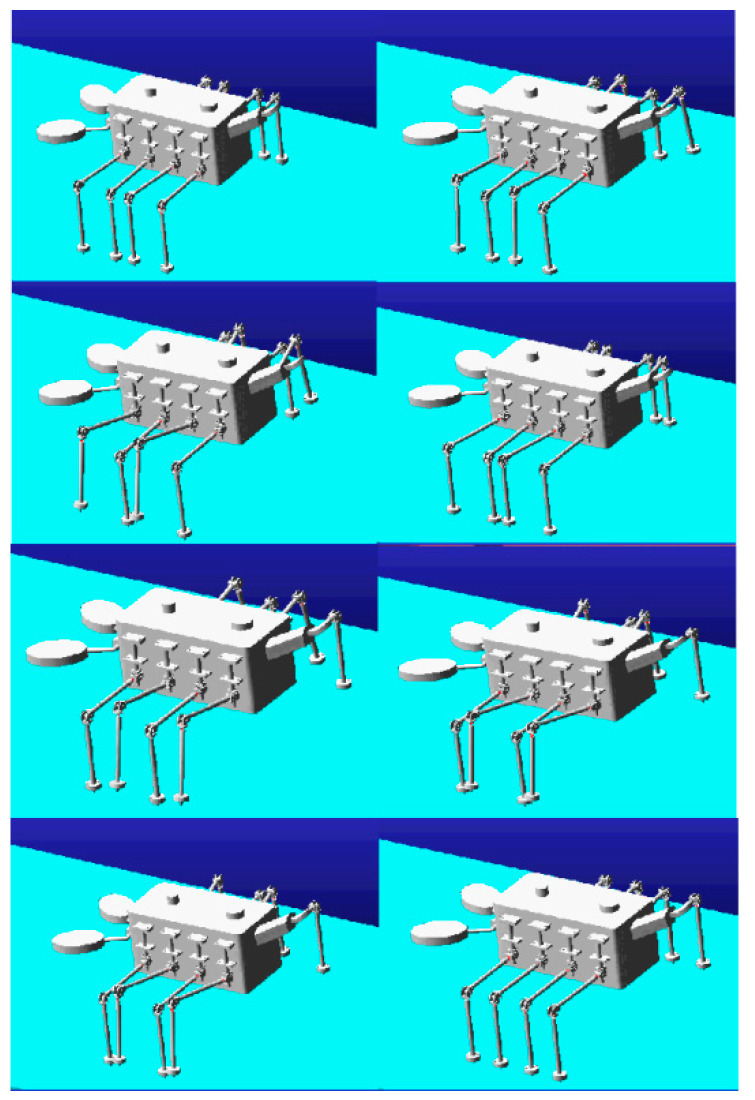
Adams simulation of machine lobster walking.

**Figure 13:**
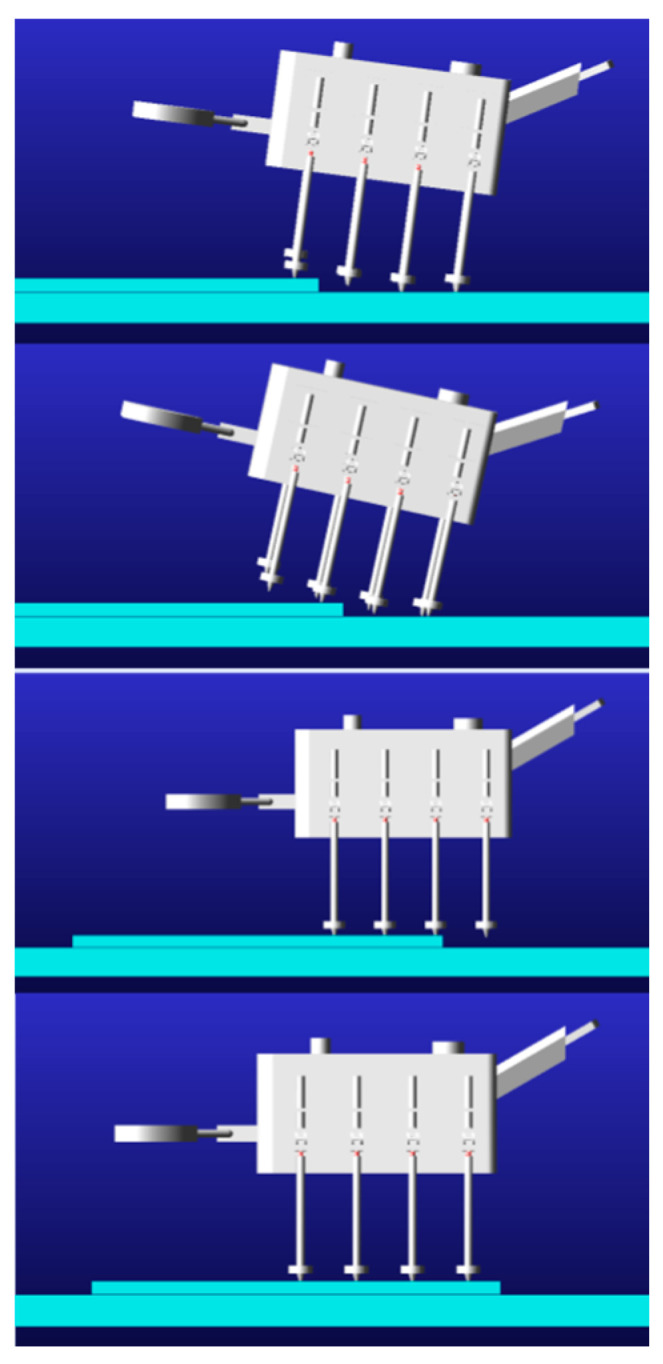
Adams simulation of robotic lobster obstacle crossing walking.

The machine lobster model was incorporated into the Adams simulation environment, in which the octopod gait was established. The subsequent simulation produced the following representation of the locomotion performance of the machine lobster: As depicted in Figure 12, the machine lobster exhibited stable locomotion via an octopod gait at ground level. As shown in Figure 13, the robotic lobster can cross obstacles on stairs.

## 5. Conclusions

This study was inspired by biological lobsters to design a machine lobster for underground and mining environments, featuring a leg structure with three degrees of freedom. Compared to the complex structures of previously designed eight-legged robots, the machine lobster offers a more streamlined and efficient design. The legs and torso are interconnected and controlled using shape memory alloys, facilitating a biomimetic monopodial and polypoidal gait specifically tailored for the machine lobster’s 24 coupled degrees of freedom. The feasibility of this gait was confirmed through simulations conducted in MATLAB. Additionally, a multi-layered grid central pattern generator (CPG) neural network was developed for controlling the machine lobster. Unlike other generators, the one selected in this study exhibited fast convergence and strong robustness. Simulations conducted in Simulink have validated its rapid convergence and robust stability, demonstrating the network’s effective and reliable capability in controlling the proposed gait. Currently, the research presented in this article requires further improvement and enhancement. The following points will be addressed for future optimization:

(1) The machine lobster must enhance the forward and backward gaits while developing supplementary gaits to fully utilize the octopodal structure and three degrees of freedom of the machine lobster legs. We plan to develop a turning gait using a differential steering mechanism. This involves adjusting the phase difference between the left and right central pattern generator (CPG) networks to generate rotational torque. Additionally, we will incorporate shape memory alloy (SMA)-driven tail deflection and clamp limb assistance to reduce the turning radius. Simultaneously, we aim to enhance the multi-level adaptability of both forward and backward gaits by dynamically adjusting step frequency and joint amplitude based on LiDAR terrain scanning and inertial measurement unit (IMU) attitude feedback. This approach will enable three operational modes: low-speed with high stability, medium-speed cruising, and high-speed breakthroughs.
(2) Refinement of the oscillator mathematical model and adjustment of the central pattern generator (CPG) neural network architecture are necessary to accommodate a broader spectrum of gaits.
(3) The refinement of the oscillator mathematical model and the adjustment of the central pattern generator (CPG) neural network architecture are essential for accommodating a wider range of gaits. Integrating a feedback mechanism into the robotic lobster to adapt its locomotion based on environmental inputs is crucial. We plan to enhance the adaptability of bio-inspired robotic lobsters in complex underground environments, wherein a hierarchical feedback control architecture is proposed. First, real-time perception of terrain slope, ground reaction forces, and obstacle information will be achieved using body inertial measurement units (IMUs), foot force sensors, and laser radars. This will enable dynamic adjustments to the oscillation frequency, phase difference, and joint movement amplitude of the high-level CPG network (e.g., reducing step frequency or adjusting stance duration to prevent overturning based on slope).

## Figures and Tables

**Table 1:** DH table for leg structures.

Rod i	Rod Length $a_{i-1}/mm$	Rotation Angle $\alpha_{i-1}/rad$	Joint Distance $d_{i}/mm$	Joint Variable $\theta_{i}/rad$
1	0	0	0	$\theta_{1}$
2	0	π/2	$d_{1}$	$\theta_{2}$
3	$L_{1}$	0	0	$\theta_{3}$

**Table 2:** Physical parameters of machine lobster leg structures.

Physical Parameter	$L_{B}$	$W_{B}$	$H_{B}$	$d_{1}$	$L_{1}$	$L_{2}$
Sizes/mm	300	150	150	75	185	191.5

## Data Availability

The original contributions presented in this study are included in the article. Further inquiries can be directed to the corresponding author.
